# BRC4Env, a network of Biological Resource Centres for research in environmental and agricultural sciences

**DOI:** 10.1007/s11356-018-1973-7

**Published:** 2018-04-19

**Authors:** Christian Mougin, Emmanuelle Artige, Frédéric Marchand, Samuel Mondy, Céline Ratié, Nadine Sellier, Philippe Castagnone-Sereno, Armelle Cœur D’Acier, Daniel Esmenjaud, Céline Faivre-Primot, Laurent Granjon, Valérie Hamelet, Frederic Lange, Sylvie Pagès, Frédéric Rimet, Nicolas Ris, Guillaume Sallé

**Affiliations:** 1 UMR ECOSYS, INRA, AgroParisTech, Université Paris-Saclay, 78026 Versailles, France; 2 UMR CBGP, INRA, CIRAD, IRD, Montpellier SupAgro, 34988 Montferrier-sur-Lez, France; 3 UE U3E, INRA, pôle AFB-INRA Gest’Aqua, 35042 Rennes, France; 4 Agroécologie, AgroSup Dijon, INRA, Univ. Bourgogne Franche-Comté, F-21000 Dijon, France; 5 US InfoSol, INRA, 45075 Orléans, France; 6 INRA, Université Côte d’Azur, CNRS, ISA, 06900 Sophia Antipolis, France; 7 UMR CARRTEL, INRA, Université de Savoie, 74203 Thonon-les-Bains, France; 8 UMR ECOBIOP, INRA, Université Pau & Pays Adour, pôle AFB-INRA Gest’Aqua, 64310 Saint-Pee-sur-Nivelle, France; 9 UMR DGIMI, INRA, Université de Montpellier, 34095 Montpellier, France; 10 UMR ISP, INRA, Université François Rabelais, 37380 Nouzilly, France

**Keywords:** Genomic, Genetic, Biological, Resource, Ecosystem, Research, Environment, Agriculture, Platform, Infrastructure

## Abstract

The Biological Resource Centre for the Environment BRC4Env is a network of Biological Resource Centres (BRCs) and collections whose leading objectives are to improve the visibility of genetic and biological resources maintained by its BRCs and collections and to facilitate their use by a large research community, from agriculture research to life sciences and environmental sciences. Its added value relies on sharing skills, harmonizing practices, triggering projects in comparative biology, and ultimately proposing a single-entry portal to facilitate access to documented samples, taking into account the partnership policies of research institutions as well as the legal frame which varies with the biological nature of resources. BRC4Env currently includes three BRCs: the Centre for Soil Genetic Resources of the platform GenoSol, in partnership with the European Conservatory of Soil Samples; the Egg Parasitoids Collection (EP-Coll); and the collection of ichthyological samples, Colisa. BRC4Env is also associated to several biological collections: microbial consortia (entomopathogenic bacteria, freshwater microalgae…), terrestrial arthropods, nematodes (plant parasitic, entomopathogenic, animal parasitic...), and small mammals. The BRCs and collections of BRC4Env are involved in partnership with academic scientists, as well as private companies, in the fields of medicinal mining, biocontrol, sustainable agriculture, and additional sectors. Moreover, the staff of the BRCs is involved in many training courses for students from French licence degree to Ph.D, engineers, as well as ongoing training.

## Introduction

Conserving and ensuring the availability of genetic and biological resources, particularly for the purposes of research, conservation of biodiversity, education, and economic valorization, are today major societal and scientific challenges, mainly faced by Biological Resource Centres (BRCs). In that context, AgroBRC (Agronomic Resources for Research RARe, http://www6.inra.fr/agrobrc-rare/) is a distributed Research Infrastructure connecting French BRCs which are maintaining genetic and biological resources produced and characterized by research on domestic animals, crops and model plant species, wild relatives of domestic species, forestry, micro-organisms relevant for agronomy, food science and environment, soil microbial consortia, and invertebrates and vertebrates of the environment. The BRCs of AgroBRC-RARe are grouped in five pillars according to the origin of their resources: animal/crop/forestry/micro-organisms/environment. AgroBRC-RARe is registered on the French national roadmap for Research Infrastructures.

Since late 2015, the pillar “Biological Resource Centre for the Environment” BRC4Env (https://www.brc4env.fr/) is a network of BRCs and collections hosted by several French research institutions INRA (Institut National de la Recherche Agronomique), IRD (Institut de Recherche et Développement), CIRAD (Centre de Coopération Internationale en Recherche Agronomique pour le Développement), and CNRS (Centre National de la Recherche Scientifique), as well as associated to technical and higher education institutions. The leading objective of BRC4Env is to improve the French and European visibility of biological resources maintained by its BRCs and to facilitate their use by a large research community, from agriculture research to life sciences and environmental sciences. Its added value relies on sharing skills, harmonizing practices, triggering projects in comparative biology, and ultimately proposing a single-entry portal to facilitate access to documented samples, taking into account the partnership policies of research institutions as well as the legal frame which varies with the biological nature of resources (i.e., the implementation of the Nagoya protocol).

**Fig. 1:**
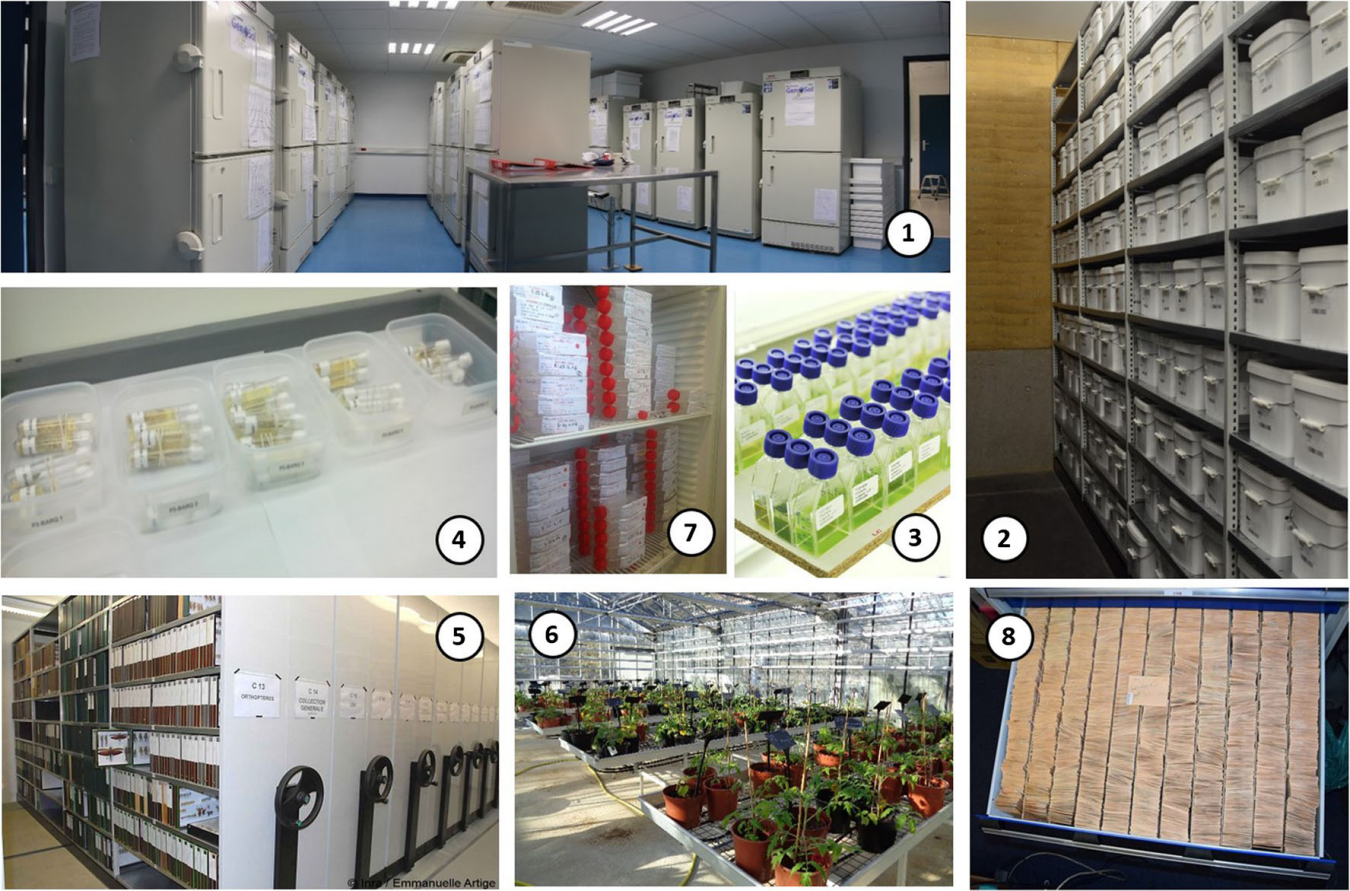
Views of the BRCs and collections of the network BRC4Env. (1) Centre for Soil Genetic Resources GenoSol, (2) European Conservatory of Soils Samples, (3) Freshwater microalgae, (4) Egg Parasitoids Collection, (5) Terrestrial arthropod and small mammals collection, (6) Plant-parasitic nematodes, (7) Entomopathogenic nematodes, (8) Collection of ichthyological samples Colisa

The biological and/or genomic resources of BRC4Env are sampled from soils, sediments, waters, natural, and agricultural ecosystems... and preserved in organized collections. They include microbial consortia and animal resources (invertebrates and vertebrates). Currently, BRC4Env includes three BRCs: the Centre for Soil Genetic Resources of the platform GenoSol in partnership with the European Conservatory of Soils Samples, the Egg Parasitoids Collection EP-Coll, and the collection of ichthyological samples, Colisa. BRC4Env also comprises other biological collections including microbial consortia (entomopathogenic bacteria, freshwater microalgae…), terrestrial arthropods (insects and mites), nematodes (plant parasitic, entomopathogenic, animal parasitic...), and small mammals (Table 1). These BRCs and collections have collected more than 1,220,000 samples to date. They possess the structures and equipment to maintain, characterize, and distribute all their biological resources for end-users (Fig. 1).

**Table 1:** Biological Resource Centres and collections of the network BRC4Env

CRB/collections	Samples	Species	Contact
Network BRC4Env			C. Mougin, UMR ECOSYS
Environmental matrices			
Centre for Soil Genetic Resources GenoSol (BRC)	14,000 extracts of soil DNA	Microbial consortia (metagenomes)	S. Mondy, C. Faivre-Primot, UMR Agroecologie
European Conservatory of Soils Samples	47,000 dry soil samples	/	C. Ratié, US InfoSol
Microbial consortia			
Entomopathogenic bacteria	650 bacterial strains from the 5 continents Strains stored in glycerol, or freezed at − 80 °C or liquid nitrogen	27 species of *Xenorhabdus* 4 species of *Photorhabdus*	S. Pagès, UMR DGIMI
Freshwater microalgae	947 strains among diatomophyceae, chlorophyceae… 486 living cultures	296 taxa (species/varieties)	F Rimet, UMR CARRTEL
Invertebrates			
Arthropodes			
Egg Parasitoids Collection EP-Coll (BRC)	130 living strains of trichogrammae 2000 samples of DNA	15 living species 25 species as DNA	N. Ris, N. Sellier, UMR ISA
Terrestrial Arthropod Collection	1,000,000 specimens of insects, stored dry, in ethanol or in slides 10,000 DNA 20,000 specimens of mites in slides, or in ethanol 1000 samples of DNA	60,000 species for various groups of high agronomic importance in Europe (phytophagous, forest and auxiliary arthropods): aphids, dipterous leafminers, (*Agromyzidae*), thrips, sawflies, chalcidid, leafrollers, moths and *Neuroptera*, *Phytoseiidae* mites and houses large series of specimens from the Tropics	E. Artige, A. Cœur-D’Acier, UMR CBGP
Nematodes			
Plant-parasitic nematodes	145 living isolates maintained in glasshouse on plant (*Meloidogyne* spp. and *Xiphinema* spp.) or in Petri dishes on fungi (*Bursaphelenchus* spp.)	80 isolates of 10 species of *Meloidogyne* spp. 30 isolates of 4 species of *Xiphinema* spp. 35 isolates of 10 species of *Bursaphelenchus* spp.	P. Castagnone-Sereno, D. Esmenjaud, UMR ISA
Entomopathogenic nematodes	140 living strains stored in dry media (100 strains of *Steinernema* and 40 strains of *Heterorhabditis)*	26 species of *Steinernema* 3 species of *Heterorhabditis*	S. Pagès, UMR DGIMI
Animal parasitic nematodes	9435 samples including male and female adults and infective larvae	31 parasitic species of 11 host species	G. Sallé, UMR ISP
Vertebrates			
Small Mammals collection	81,000 samples 2250 whole specimens (skins, skulls 10,000 skulls, 4000 skins 50,000 tissues in ethanol 2000 DNA extracts 5500 buffered RNA 500 cell cultures 300 chromosome preparations 5000 nematodes endoparasites in ethanol 1500 ectoparasites (fleas, ticks, mites) in ethanol	*Cricetidae*, *Arvicolinae* subfamily (France: *Microtus*, *Arvicola*, *Myodes*), *Muridae* mostly *Murinae* (Europe, Africa, Asia: *Apodemus*, *Mus*) and *Gerbillinae* (West and North); some specimen of *Sciuridae*, *Nesomyidae* (*Dendromurinae*) and *Dipodidae*. *Soricomorpha* mostly *Soricidae* and *Crocidurinae* (*Sorex*, *Neomys*, *Suncus*) Some *Erinaceomorpha* (*Erinaceidae*, *Erinaceinae*) Some *Chriroptera*	E. Artige, L/ Granjon, UMR CBGP
Collection of ichthyological samples Colisa (BRC)	200,000 scales, pieces of fin, otoliths and caps of freshwater fish and lamprey	26 species collected in whole France during 46 years	F. Marchand, UE U3E Valérie Hamelet, UMR CARRTEL Frederic Lange, UMR ECOBIOP

The network develops interactions with analytical platforms for the characterization of samples (https://www.brc4env.fr/Associated-platforms) in biochemistry: Biochem-Env in Versailles (Cheviron et al. 2018); environmental chemistry: Platinaae in Arras and Bordeaux; genomics: GenoSol; and imaging: DimaCell in Dijon. In Sophia, the BRCs and collection are line with the platform SPIBOC (microscopy, analytical biochemistry, bioinformatics, genomics). BRC4Env can also access to ecosystems through two networks: AnaEE-France (Mougin et al. 2015) and Recotox (https://www.recotox.eu/recotox_eng/, Mougin et al., this Special Issue).

### Focus on the BRCs of BRC4Env

The Centre for Soil Genetic Resources (CSGR, https://www.brc4env.fr/BRCs-and-collections/Environmental-matrices/GenoSol) of the platform GenoSol provides a national BRC for soils unique in Europe for the storage and conservation of genetic resources (DNA) and making them available to the scientific community. Most of the stored soil samples come from soil survey networks (at national, regional, and landscape scales), environmental research observatories (from INRA, CNRS, and University), long-term experimental sites (within French and European networks), and networks of farms. All these sites are involved in scientific questions concerning the environmental assessment of soil uses and management, as well as the impact of global changes. The platform GenoSol is a strategic tool to supply these sites in the long term and monitor soil biodiversity.

The CSGR of GenoSol is hosted by the research department UMR1347 Agroecologie in Dijon, France. It offers sampling and storage facilities (sampling material, freezers, and dedicated laboratory and rooms) with the possibility to store soils from non-European countries granted by a quarantine agreement. The GenoSol platform has all the laboratory facilities needed for molecular biology studies in the service “Laboratory of Molecular Analysis and Development.” Indeed, the CSGR will provide DNA extracted from soil sample rather than soil itself, as soil quantity is limited (ca 50 g per sample). In consequence, the CSGR of GenoSol needs to routinize and automatize DNA extraction from sample in order to provide good-quality DNA.

GenoSol performs its activities in partnership with the European Conservatory of Soils Samples (CEES, https://www.brc4env.fr/BRCs-and-collections/Environmental-matrices/CEES) hosted by the US1106 InfoSol in Orleans, in the frame of the French Network of Measurement of Soil Quality (RMQS, Réseau de Mesures de la Qualité des Sols). The CEES stores an archive of French soils collected within the national soil survey and monitoring programs coordinated by the French Soils Scientific Interest Group (GIS Sol). This collective infrastructure is also supporting French and European research programs and Research Infrastructures such as ICOS, with inputs in expertise and support on sampling strategies and methods, sample collection, preparation and archiving, supply of samples to research programs, and partner analysis laboratories. The first sampling campaign of RMQS occurred from 2000 to 2009 and corresponded to 20,000 samples from 2200 sites. The second sampling campaign began in 2016 and will finish in 2027.

In order to open widely the conservatory to research, GenoSol platform aims to establish a convention with the soil owners. We aimed to provide an open storage that allows researchers to access the genetic resources (with the establishment of a Material Transfer Agreement) or restricted storage (access restricted to the soil owner).

The Egg Parasitoids Collection (EP-Coll, https://www.brc4env.fr/BRCs-and-collections/Invertebrates/Arthropods/EP-Coll) aims to collect, characterize, maintain, investigate, and share egg parasitoids’ strains, these organisms being useful for biological control, applied entomology, and evolutionary ecology.

In addition to (i) the conservation of living collections and (ii) the management of strains’ transfers, the activities of the BRC are (iii) the collection of new strains (species and/or populations); (iv) the characterization of these individuals using morphological, molecular, phenotypic, and ecological criteria; (v) the optimization of sampling, rearing, and management methods; and (vi) the optimization of phenotyping methods.

The BRC is deposited at the UMR1355 Institut Sophia AgroBiotech (ISA) in Sophia Antipolis, France. ISA is a research laboratory which benefits of an original collective scientific infrastructure PlantBIOs (Plants Biostimulation and Biocontrol facilities and expertise (https://www6.paca.inra.fr/institut-sophia-agrobiotech/Infrastructure-PlantBIOs)), with greenhouses and controlled environment chambers for carrying out in vitro and in situ experiments, with official agreement for the manipulation of quarantine insects and nematodes, and all the laboratory needed for morphological and molecular biology studies.

For the BRC EP-Coll, the main technological/methodological developments rely on integrative taxonomy combining molecular and morphological information as well as crossing experiment, improvement of sampling through various methods (sentinel eggs with various eggs, sampling of natural egg clutches, etc.), and thermal biology including the development of a prototype for the evaluation of some key parameters.

The historical collection of fish scales and tissues (Colisa, https://www.brc4env.fr/BRCs-and-collections/Vertebrates/Colisa) results from the merging of historical samples collected by three INRA units (U3E in Rennes, ECOBIOP in Saint-Pée-sur-Nivelle, and CARRTEL in Thonon-les-Bains) and by FBA (French national Agency for Biodiversity) as part of the AFB-INRA Pole “Gest’aqua.” These samples come from long-term monitoring and research activities conducted by these units and from a national catch declaration scheme (angling and professional fishery).

Beyond age and growth, these samples, carrying DNA, allow genetic characterization of individuals or population. Using microchemistry, these samples allow to characterize the trophic status and the environmental conditions of life of these animals. The conservation of these tissues allows investigating historical changes (global and local) that have occurred.

### BRC4Env as a relevant support for research programs

The network BRC4Env and its BRCs and collections implement a quality management according to the INRA Quality Framework v2 for optimized functioning, continual improvement, and stakeholder satisfaction. They plan to move to national (NF S 96-900) or international (ISO 9001:2015) certification in the next 2 years. Numerous BRCs and collections ensure the traceability and planning of their animation activities using the software AQ-tools developed by CIRAD (http://golo.cirad.fr). The network BRC4Env, the CSGR of GenoSol, EP-Coll, and Colisa have been labelled as BRCs by the French organism IBiSA.

The BRCs follow the rules of good scientific and ethical practices, policies, and regulations. BRC4Env is committed to provide an operational support to its members regarding the implementation of the Access and Benefit Sharing and the Nagoya protocol, and the staff of the BRC follows training accordingly. Several BRCs benefit from a French approval of quarantine facilities, for handling and experiments on soils and organisms from non-continental European territories and non-European countries.

Thus, the BRCs and collections of BRC4Env are involved in partnership with academic scientists, as well as with private companies, in the fields of medicinal mining, biocontrol, sustainable agriculture, and additional sectors. Since their creation, the BRCs of BRC4Env have been involved in about 140 research contracts as partners of academic laboratories, and 30 are ongoing during the year 2016, at French or international scales. The projects are led by scientists belonging to the departments hosting the BRCs, or by external partners.

Examples or research projects involving BRC4Env collections are presented below.

1. The environmental assessment of soil uses and management, as well as the impact of global changes on soil biodiversity, positioning BRC4Env as a strategic tool to supply the long-term sites for observation and experimentation. In that respect, the Centre for Soil Genetic Resources of the platform GenoSol collects, stores, and characterizes soil samples from the different sites of the SOERE PRO (Systèmes d’observation et d’expérimentation au long terme pour la recherche en Environnement – Produits résiduaires Organiques), by providing temporal set of samples to scientists (Obriot et al. 2016). In addition, the project CammiSole aims at understanding the role of soil microbial biodiversity in the provision of services of plant production, nutrient cycling, and carbon sequestration, in relationships to global changes in soils from Madagascar (Razanamalala et al. 2018). The project ECOMIC-RMQS (Réseau de Mesures de la Qualité des Sols) aims at better defining and understanding the processes governing the microbial biodiversity of soils, by a better estimation and characterization of the “beta” diversity of the communities (species turnover along an environmental gradient). The RMQS1 network offers 2200 sampled soils and covers the whole French territory (Ranjard et al. 2013; Terrat et al. 2017; Horrigue et al. 2016). The RMQS2 program is ongoing (Swiderski et al. 2017).
2. The freshwater microalgae culture collection of the BRC4Env (Thonon Culture Collection) holds more than 900 strains (Diatom, Chlorophyceae, Cyanobacteria…). Microalgae and especially Diatoms are known to be excellent indicators of aquatic ecosystem pollution (Rimet 2012). This collection enabled to develop a reference barcoding library R-Syst:diatom (Rimet et al. 2016) which enabled to set up new tools based on DNA metabarcoding to assess river and lake quality which are more efficient than classical microscope-based methods (e.g., Keck et al. 2017). Such methods are currently standardized at European level.
3. Trichograms of the BRC EP-Coll are relevant biological models for eco-evolutionary investigations and constitute a reservoir of candidate strains (species/populations/genotypes) for biocontrol (Marchand et al. 2017; Martinez-Rodriguez et al. 2016). The ANR program TRIPTIC (*Trichogramma* pour la protection des cultures: Pangénomique, Traits d’histoire de vIe et Capacités d’établissement) aims at characterizing the inter- and intra-specific diversity of the *Trichogramma* species at several levels of investigation (genome, individual, population (Dumbardon et al. 2018) and even community) with explicit outputs for both basic (phylogeny, systematics, comparative analysis; Ris et al. 2016), applied (potential strains of biocontrol agents), and regulation (native versus exotic biodiversity) issues. Complementary programs such as the FP7/PEOPLE/IAPP COLBICS aim at more readily exploiting these inter- and intra-specific diversities for the development of new biological control agents (Declaration of Invention “DI RV 17 0076: Genetic improvement of *T. brassicae*”).
4. The Terrestrial Arthropod Collection of BRC4Env is the basis of various collaborative national and international research programs related to plant health or biodiversity, combining molecular, morphological, and chorological information. This collection, managed by the CBGP (Artige 2017), is used for several molecular barcoding programs (Rasplus and Loomans 2017).

The key action Sys3D (Systematics for the Detection, the Diagnosis and the iDentification; whose goal is to create fast and reliable tools for plant diagnosis) of the INRA metaprogram SMaCH (Sustainable Management of Crop Health, 2010–2020) relies on a part of this Arthropods Collection: sequenced specimens, studied morphologically, preserved with their DNA for traceability purposes (Laval and Streito 2017).

The reference collection of European quarantine Arthropods, an outcome of the European project FP7/Q-BOL/Q-Bank (Rasplus and Loomans 2017) is also hosted at CBGP. Informative genes from species selected in EU Directive and EPPO lists are DNA barcoded from vouchered specimens. Methodological outcomes of this program are a non-destructive extraction technique, an amplification protocol using primers multiplexing, a bioinformatics pipeline for sequences validation, and a structured database with a web interface. This website is automatically updated using the DBMS (Database Managing System) used for the Terrestrial Arthropods collection.

New species have recently been described using specimens from this collection. Examples include a phytophagous mite (Arabuli et al. 2016), a seed pest beetle (Mouttet et al. 2016), or a new species of darkling beetle (Soldati et al. 2016). The collection is also used by research programs aiming to estimate the relative weights of ecological factors and geographical isolation on the speciation processes within several groups of Arthropods (Meseguer et al. 2015; Migeon 2015).

In return, this collection is regularly enriched by specimens collected via these research programs.

5) The samples of the collection Colisa, carrying DNA, allow genetic characterization of wild fish individuals or population. Via microchemistry, these samples allow to characterize the trophic status and the environmental conditions of life of these animals (Rougemont et al. 2016). The conservation of these tissues allows investigating historical changes (global and local) that have occurred. The project “Temporal evolution of genetic parameters in Lake Geneva’s arctic charr population” assess the spatial and temporal evolution of genetic parameters charr population (Savary et al. 2017). Atlantic Aquatic Resource Conservation (AARC) was a collaborative effort between several European institutions, and combined high-quality science with practical help for rivers around the Atlantic coast. This was achieved by working at the appropriate scale to the problem in hand, using a variety of different approaches; including conservation genetics (historical tissue collection), river restoration, aquaculture, and educational approaches across the European partners (Quéméré et al. 2016).
6) The Plant-parasitic Nematodes Collection of BRC4Env has been the support of various collaborative European research programs. For example, the pinewood nematode collection has been instrumental in the achievement of some objectives of the FP7/EU-project REPHRAME (Development of improved methods for detection, control and eradication of pine wood nematode in support of EU Plant Health Policy). Together with additional field sampling, these biological resources have been used to characterize the genetic diversity of nematode populations from both the native and the invaded areas, and to infer the worldwide routes of invasion of the parasite (Vieira et al. 2014; Mallez et al. 2015; Ciordia et al. 2016). These new data are of direct practical importance for the implementation of efficient phytosanitary measures for quarantine regulation.
7) The collection of animal parasitic nematodes has been established 30 years ago. It has originally been dedicated to ruminant trichostrongylid to delineate the genetic basis of anthelmintic resistance (Silvestre and Humbert 2002; Neveu et al. 2007). Recent surveys provided additional samples for equine parasite species (Sallé et al. 2017) that was also used for work on anthelmintic drug receptors (Courtot et al. 2015). The breadth of its sampling area has recently been used for a project dedicated to characterize the global diversity of *Haemonchus contortus* populations, one of the most deadly livestock parasitic nematodes (Marie-Curie FP7 COFUND People Programme, grant agreement 609398). The molt of this collection towards a more formal BRC has been recently initiated.
8) The CBGP Small Mammal Collection, including specimens (skins and/or skulls) and their corresponding samples (i.e., organ parts, blood or cells in ethanol, various buffers, or cryopreserved), and the Small Mammal Database gathering all the associated information have been the support of a number of studies over the last years. Among others, we may cite the description of the invasion of Senegal by the house mouse (Dalecky et al. 2015) based on the compilation of data on commensal rodents in Senegal over the period 1983–2014 in the frame of the JCJC ANR programme “ENEMI”; a review on camouflage in a set of species of gerbil species based on analyses of their skin reflectance (Boratyński et al. 2017); various studies on the phylogeny of supraspecific groups (Ndiaye et al. 2012, 2016…) or intraspecific phylogeography (Hima et al. 2011; Dobigny et al. 2013); and a growing amount of works highlighting the role of small mammals as reservoirs of a number of zoonotic pathogens potentially transmissible to humans (e.g., Dobigny et al. 2015 for *Leptospira*, Tatard et al. 2017 for *Trypanosoma*, Diagne et al. 2017a, b for bacteria and viruses in West Africa). This collection and associated database have represented the basic materials for an updated synthesis on West African rodents (Granjon and Duplantier 2009), and also serve as depository of information for the “West African Observatory on small mammals as indicators of environmental changes” (ObsMiCE), relying on a network of observation sites all over West Africa, and which is currently supported by IRD and OSU Pytheas fundings.

More than 200 scientific publications have been produced by the BRCs of the network since their creation. Book chapters, presentations at conferences, and technical notes have been also produced. Moreover, the staff of the BRCs is involved in many training courses for students from French license to Ph.D degree, engineers, as well as ongoing training. They welcome also numerous students for internships every year.

### The next steps of BRC4Env

BRC4Env builds on existing facilities, equipment, and human resources of its BRCs, collections, and hosting laboratories. The vision of BRC4Env is the long-term collaboration and common development of strategies, best practices, and standards related to the use and preservation of environmental resources for basic and applied research in the field of environment and agronomy. Achieving fully transparent collection access procedures, securing financial sustainability and ensuring conformity to regulation form critical objectives for BRC4Env. To reach these objectives, BRC4Env will also benefit from the know-how developed by the other pillars of AgroBRC-RARe, which have been established for a longer time.

In the context of the project “Implementing ABS regulations by agronomic BRCs in the national infrastructure RARe” (ABS4BRCs), BRCs and collections of BRC4Env will determine the legal status of their accessions and consequences for their introduction and distribution, regarding the Access and Benefit Sharing as well as French and European policies. A case study based on soil samples will be developed during the project, because these resources are not well characterized at the time of sampling in terms of taxonomy.

The main objective of BRC4Env is to develop the Information System of the network, currently constituted by shared tools and distributed services among BRCs, by making these tools interoperable and relevant regarding the Nagoya policy. A specificity of BRC4Env is the hosting of living collections of invertebrates. The Information System must provide and share information concerning sampling, rearing, phenotyping and molecular data, and barcoding. Here, we plan to set the following requirements: (i) centralization and standardization of metadata concerning the resources, (ii) secure storage and confidentiality by controlled access, (iii) tools to perform queries (in partnership with applications to be developed by AgroBRC-RARe). A common database for registering the projects hosted by the BRCs of the network will be developed.

The Information System of BRC4Env will offer shared information dedicated to the resources hosted by its BRCs. It will also promote and contribute to make easier the access, storage, archiving of datasets, and their subsequent valorization, and provide to end-users access to the productions of the network (publications, catalogues…) by subscription to News and RSS feed. Experience gained in other pillars of AgroBRC-RARe in these fields will be used in order to optimize the functional specificities of our IS and to select the most relevant technical solution.

BRC4Env shares two main other objectives in the next future.

- Internal practice harmonization. Considering the diversity of BRCs and collections, the network will encourage practice harmonization for improved management and enhanced traceability of its activities within its BRCs and make available a comprehensive set of harmonized procedures. One main objective is the introduction (if necessary), the development, and the support for a common process-based quality management system for continual improvement and stakeholder satisfaction. Taken together, these actions will facilitate the sharing of experience and the identification of best practices within the BRCs and collections of the network. Sharing experience will then make possible to define a common policy for BRC and collections functioning, charters for sample and data access, relationships between BRCs and private partners…
- Valorization of the activities of the BRCs. The network will produce scientific and perspective papers (in open-access journals), illustrating the research questions that a better and beneficial coordination between BRCs can help to tackle, as well as the scientific and technical support they can provide to end-users. The network promotes also the production by the BRCs and collections of technical papers valorizing their expertise and know-how. It will also produce and support an action plan for attending national and relevant international conferences and workshops to increase its national and international visibility.

## Conclusion

BRC4Enc is a network structuring the BRCs dedicated to environmental resources in France. It is currently seeking for similar networks of BRCs in Europe and worldwide to be a French component of international networks. Today, environmental resources are mainly taken into account in environmental specimen banking. BRC4Env can offer a frame to host samples and collections specifically addressing ecotoxicological issues.
